# Battle Against Musculoskeletal Tumors: Descriptive Data of Military Hospital Experience

**DOI:** 10.3389/fpubh.2020.00097

**Published:** 2020-03-25

**Authors:** Cagri Neyisci, Yusuf Erdem

**Affiliations:** Department of Orthopaedics and Traumatology, Gulhane Training and Research Hospital, Ankara, Turkey

**Keywords:** tumor, musculoskeletal, military, experience, benign, malignant

## Abstract

**Background:** Management of musculoskeletal tumors remains challenging for orthopedic surgeons. The aim of this cross-sectional study was to present the prevalence and localization of musculoskeletal disorders diagnosed and treated at a tertiary referral military hospital.

**Method:** A total of 552 patients' medical records who presented to our clinic between 2009 and 2014 with the diagnosis of musculoskeletal tumors were retrospectively analyzed according to age, gender, bone/soft tissue localization, histopathological diagnosis, incidence, and treatment.

**Results:** Of the cases diagnosed with tumor, 225 were soft tissue localized, 317 bone localized, and 10 tumor-like lesions. The most common primary benign soft tissue tumors were lipoma, ganglion, and giant cell tumor of tendon sheath, while the most common malignant soft tissue tumors were liposarcoma and synovial sarcoma, respectively. The most common primary bone tumors were osteochondroma, enchondroma, and simple bone cyst, while the most common malignant bone tumors were osteosarcoma, Ewing sarcoma and chondrosarcoma, respectively. Myositis ossificans was found as the most common tumor-like lesion.

**Conclusion:** Descriptive data in musculoskeletal tumors is crucial in terms of improving treatment and reducing mortality. In this study, no significant difference was found between the data of tertiary military hospital regarding epidemiology of musculoskeletal system tumors and those from the literatures around Turkey.

## Introduction

Although musculoskeletal tumors including bone and soft tissue tumors are relatively rare pathologies among total world cancer incidence, the management should compromise great attention (1). While great interest in orthopedic oncology as a subspecialty has been expanding in many countries, it remains challenging in orthopedic practice due to ignored educational opportunities (2).

More than 80% of the population of the world is located in developing countries where considerable diversity in terms of resource and expertise availability still exists in managing musculoskeletal tumors (3). Furthermore, a large number of patients in these countries are treated by inexperienced surgeons who have little knowledge and update about musculoskeletal oncology principles (4, 5).

In Turkey, there are few centers dealing with the management of orthopedic oncology. Among those centers, tertiary military hospital, is primarily responsible for the management of musculoskeletal tumors in military personnel and their families. Lack of subspecialized centers in military health system organization chart, the patients with a diagnosis of whether bone or soft tissue tumors in all around the country is referred to the tertiary military hospital.

In this report, we aimed to present 5-year data and experience of tertiary military hospital on musculoskeletal oncology among the military personnel's and their families. In addition, it is emphasized that the mission of our hospital is not only the war-related injury management, but also the musculoskeletal tumor treatment and prevention of labor loss.

## Materials and Methods

A total of 552 bone and soft tissue tumors of 552 consecutive patients who underwent musculoskeletal oncologic surgery between 2009 and 2014 were retrospectively analyzed. A written informed consent was obtained from each patient. The study protocol was approved by Gülhane Military Medical Academy Local Ethics Committee (2014-13). The study was conducted in accordance with the principles of the Declaration of Helsinki.

All patients age, gender, diagnosis, tumor localization, and treatment were analyzed. All tumoral pathologies were divided as bone, soft tissue, and tumor-like lesions, and classified in separate groups regarding upper and lower extremities with the status of benign, malignant and tumor-like lesions (Tables 1–10).

**Table 1:** Distribution of tumors in the extremity.

	**Soft Tissue**		**Bone**		**Tumor like lesions**	**Total**
	**Benign**	**Malignant**	**Benign**	**Malignant**		
Upper extremity	102	8	64	14	2	190
Male	70	6	53	8	2	139
Female	32	2	11	6	0	51
Lower extremity	89	26	195	44	8	362
Male	65	20	177	34	7	303
Female	24	6	18	10	1	59
Total	191	34	259	58	10	552
Male	135	26	230	42	9	442
Female	56	8	29	16	1	110

**Table 2:** Benign soft tissue tumors of the upper extremity.

**Diagnosis**	***N* (*n* = 102)**	**Age (Year) – (Min-Max/Median)**	**Location**	**Treatment**	**Recurrence**	**Revision**
Hemangioma	3	19–43/21	Arm	2 Excision 1 Follow-up	–	–
Giant cell tumor of the tendon sheath	37	18–45/23	30 Hand 7 Wrist	Excision	7	Excision
Lipoma	6	21–41/29	4 Shoulder 1 Arm 1 Forearm	Excision	-	-
Synovial chondromatosis	1	25	Shoulder	Excision	–	–
Aggressive fibromatosis (Desmoid tumor)	2	14–24	1 Shoulder 1 Forearm	Excision	–	–
Fibroma	1	20	Wrist	Excision	–	–
Ganglion	52	18–59/26	33 Arm 19 Wrist	Excision	3	Excision

**Table 3:** Malignant soft tissue tumors of the upper extremity.

**Diagnosis**	***N* (*n* = 8)**	**Age (Year) – (Min-Max/Median)**	**Location**	**Treatment**	**Recurrence**	**Revision**
Synovial Sarcoma	1	22	Wrist	Below-elbow amputation	–	–
Dermatofibrosarcoma Protuberans	1	23	Arm	Wide excision	–	–
Rhabdomyosarcoma	1	19	Elbow (Antecubital)	Above-elbow amputation	–	–
Liposarcoma	5	45–59/52	2 Shoulder 2 Arm 1 Forearm	Wide excision	3	2 Wide excision 1 Above-elbow amputation

**Table 4:** Benign bone tumors of the upper extremity.

**Diagnosis**	***N* (*n* = 64)**	**Age (Year) – (Min-Max/Median)**	**Location**	**Treatment**	**Recurrence**	**Revision**
Chondroblastoma	2	24–25	1 Humerus 1 Radius	Curettage + grafting	–	–
Enchondroma	16	11–43/21	Hand	Curettage + grafting	–	–
Osteochondroma	7	5–22/20	3 Scapula 2 Humerus 2 Hand	6 Excision 1 Follow-up	–	–
Aneurysmal bone cyst	7	8–37/19	3 Humerus 2 Radius 1 Ulna 1 Scapula	3 Curettage + grafting 2 Curettage + grafting + prophylactic fixation 2 Curettage + cementation	1	Curettage + cementation
Osteoblastoma	2	20–32	Humerus	Curettage + cementation	–	–
Simple bone cyst	9	6–36/21	8 Humerus 1 Ulna	8 Curettage + grafting 1 Curettage + grafting + prophylactic fixation	–	–
Osteoid osteoma	8	11–41/21	4 Humerus 2 Hand 2 Scapula	Percutaneous curettage	–	–
Fibroxanthoma	1	22	Hand	Follow-up	–	–
Giant cell tumor of bone	3	21–25/22	2 Radius 1 Ulna	2 Curettage + cementation 1 Resection + biological reconstruction + fixation	1	Curettage + cementation
Fibrous dysplasia	7	19–25/21	7 Humerus	4 Curettage + grafting + prophylactic fixation 3 Curettage + grafting	–	–
Chondromyxoid fibroma	2	20–29	Humerus	1 Curettage + grafting + prophylactic fixation 1 Curettage + grafting	–	–

**Table 5:** Malignant bone tumors of the upper extremity.

**Diagnosis**	***N* (*n* = 14)**	**Age (Year) – (Min-Max/Median)**	**Location**	**Treatment**	**Recurrence**	**Revision**
Ewing sarcoma	5	10–28/21	3 Humerus 1 Clavicula 1 Scapula	3 Biological reconstruction 2 Tumor resection prosthesis	–	–
Osteosarcoma	3	21–24/22	1 Humerus 1 Radius 1 Ulna	2 Biological reconstruction 1 Tumor resection prosthesis	1	Shoulder disarticulation
Chondrosarcoma	3	38–52/47	Humerus	Tumor resection prosthesis	1	Forequarter amputation
Epithelioid sarcoma	1	20	Radius	Biological reconstruction	–	–
Metastasis	2	55–77	Humerus	Curettage + cementation + fixation	–	–

**Table 6:** Benign soft tissue tumors of the lower extremity.

**Diagnosis**	***N* (*n* = 89)**	**Age (Year) – (Min-Max/Median)**	**Location**	**Treatment**	**Recurrence**	**Revision**
Synovial chondromatosis	5	20–23/21	4 Knee 1 Ankle	Excision	–	–
Giant cell tumor of the tendon sheath	8	21–45/25	4 Foot 3 Knee 1 Ankle	Excision	1	Excision
Hemangioma	8	15–45/20	3 Popliteal fossa 2 Thigh 2 Foot 1 Knee	5 Excision 3 Follow-up	–	–
Lipoma	21	19–63/22	8 Thigh 5 Cruris 2 Popliteal fossa 3 Foot 2 Ankle 1 Hip	Excision	–	–
Ganglion	27	21–52/36	15 Foot 3 Knee 3 Popliteal fossa 3 Cruris 3 Ankle	Excision	–	–
Morton neuroma	12	23–37/27	Foot	Excision	1	Excision
Schwannoma	1	22	Thigh	Excision	–	–
Epidermal cyst	1	21	Foot	Excision	–	–
Fibroma	2	20–28	Thigh	Excision	–	–
Pigmented villonodular synovitis	3	17–38/20	2 Knee 1 Ankle	Excision	–	–
Aggressive fibromatosis (desmoid tumor)	1	26	Foot	Excision	–	–

**Table 7:** Malignant soft tissue tumors of the lower extremity.

**Diagnosis**	***N* (*n* = 26)**	**Age (Year) – (Min-Max/Median)**	**Location**	**Treatment**	**Recurrence**	**Revision**
Liposarcoma	10	40–67/53	7 Thigh 1 Popliteal fossa 1 Cruris 1 Pelvis	8 Wide excision 2 Above-knee amputation	2	1 Wide excision 1 Above-knee amputation
Synovial Sarcoma	9	20–45/24	1 Pelvis 4 Hip 2 Knee 1 Ankle 1 Foot	3 Tumor resection prosthesis 3 Wide excision 1 Below-knee amputation 1 Hip disarticulation 1 Hemipelvectomy	2	1 Above-knee amputation 1 Below-knee amputation
Clear cell sarcoma	1	55	Cruris	Wide excision	–	–
Kaposi sarcoma	1	75	Ankle	Below-knee amputation	–	–
Rhabdomyosarcoma	1	6	Thigh	Wide excision	–	–
Fibrosarcoma	3	20–65/24	1 Hip 1 Thigh 1 Cruris	2 Wide excision 1 Hip disarticulation	–	–
Adrenocortical carcinoma metastasis	1	2	Thigh	Wide excision	–	–

**Table 8:** Benign bone tumors of the lower extremity.

**Diagnosis**	***N* (*n* = 195)**	**Age (Year) – (Min-Max/Median)**	**Location**	**Treatment**	**Recurrence**	**Revision**
Osteochondroma	49	12–37/21	29 Femur 12 Tibia 1 Calcaneus 3 Metatarsal 2 Pelvis 2 Talus	45 Excision 4 Follow-up	–	–
Fibrous dysplasia	16	19–36/23	8 Femur 8 Tibia	9 Curettage + grafting 4 Curettage + grafting + prophylactic fixation 3 Follow-up	–	–
Giant cell tumor of bone	14	20–37/25	8 Femur 6 Tibia	11 Curettage + cementation 3 Curettage + cementation + prophylactic fixation	2	Curettage + cementation
Aneurysmal bone cyst	15	6–38/24	6 Femur 3 Pelvis 1 Patella 1 Tibia 1 Fibula 3 Calcaneus	13 Curettage + grafting 2 Curettage + grafting + prophylactic fixation	1	Curettage + grafting
Osteoblastoma	4	12–21/20	1 Femur 1 Tibia 1 Calcaneus 1 Talus	Curettage + grafting	–	–
Simple bone cyst	23	19–38/28	9 Femur 2 Tibia 8 Calcaneus 1 Patella 1 Acetabulum 2 Talus	18 Curettage + grafting 4 Curettage + grafting + prophylactic fixation 1 Follow-up	–	–
Osteoid osteoma	39	14–38/22	19 Femur 17 Tibia 1 Fibula 1 Talus 1 Metatarsal	Percutaneous curettage	–	–
Enchondroma	13	20–46/30	7 Femur 2 Tibia 1 Pelvis 1 Talus 2 Phalanx	6 Curettage + grafting 1 Curettage + grafting + prophylactic fixation 6 Follow-up	–	–
Chondroblastoma	7	19–34/21	3 Femur 3 Tibia 1 Talus	6 Curettage + grafting 1 Curettage + cementation	–	–
Non-ossifying fibroma	2	21–25	Femur	1 Curettage + grafting 1 Follow-up	–	–
Intraosseous lipoma	5	21–31/27	Calcaneus	Curettage + grafting	–	–
Fibrous cortical defect	5	21–29/21	3 Femur 2 Tibia	3 Follow-up 2 Curettage + grafting	–	–
Chondromyxoid fibroma	1	23	Femur	Curettage + grafting	–	–
Intraosseous hemangioma	1	49	Tibia	Curettage + grafting	–	–
Eosinophilic granuloma	1	21	Iliac wing	Curettage + grafting	–	–

**Table 9:** Malignant bone tumors of the lower extremity.

**Diagnosis**	***N* (*n* = 44)**	**Age (Year) – (Min-Max/Median)**	**Location**	**Treatment**	**Recurrence**	**Revision**
Osteosarcoma	20	5–34/20	14 Femur 4 Tibia 2 Pelvis	15 Tumor resection prosthesis 2 Biological reconstruction 2 Hemipelvectomy 1 Hip disarticulation	3	2 Total femur tumor resection prosthesis 1 Above-knee amputation + 2 Arthrodesis (due to 1 osteomyelitis + 1 non-union)
Metastasis	8	34–68/58	7 Femur 1 Humerus	5 Curettage + cementation + fixation 2 Cemented partial hip prosthesis 1 Tumor resection prosthesis	–	–
Ewing sarcoma	7	11–22/14	2 Iliac wing 2 Femur 1 Tibia 1 Fibula 1 Glenoid	3 Biological reconstruction 3 Tumor resection prosthesis 1 Wide resection	–	–
Chondrosarcoma	6	19–54/40	3 Pelvis 2 Femur 1 Acetabulum	3 Tumor resection prosthesis 2 Biological reconstruction 1 Hemipelvectomy	1	Wide resection
Multiple myeloma + plasmacytoma	2	30–85	Femur	1 Curettage + cementation 1 Cemented partial hip prosthesis	–	–
Fibrosarcoma	1	22	Iliac wing	Custom-made tumor resection prosthesis	–	–

**Table 10:** Tumor like lesions.

**Diagnosis**	***N* (*n* = 10)**	**Age (Year) – (Min-Max/Median)**	**Location**	**Treatment**	**Recurrence**	**Revision**
Myositis ossificans	7	19-52/22	1 Arm 1 Wrist 2 Hip 2 Thigh 1 Cruris	Excision	–	–
Brodie abscess	2	21-46	1 Fibula 1 Tibia	Curettage	–	–
Cyst hydatid	1	67	Iliac wing	Excision + application of hypertonic solution	–	–

Metastasis were considered as malignant tumors and classified in the tables of malignant tumors.

Biopsies of tumors were performed by two clinicians primarily responsible for the diagnosis of musculoskeletal tumors. No complications were encountered during or after procedures. When the diagnosis was malignant or metastasis, patients were consulted medical and radiation oncology departments whether they would receive neoadjuvant chemotherapy or radiotherapy to obtain smaller mass or prevent spreading. In need of neoadjuvant care, the definitive surgeries were scheduled after chemo/radiotherapies.

There is no difficulty in accessing chemotherapy drugs in our country where routine treatments were given by medical and radiological oncologist according to the diagnosis.

### Statistical Analysis

Statistical analysis was performed using the IBM SPSS for Mac version 23.0 software (IBM Corporation, Armonk, NY). Descriptive data were expressed in minimum, maximum, median and number and frequency.

## Results

Of the patients, 110 (20%) were female and 442 (80%) were male. Of the tumoral lesions 190 (34%) were localized in the upper extremity from shoulder joint to fingertip of hand, 362 (66%) in the lower extremity from pelvic region to fingertip of foot (Table 1).

Of all cases, 225 (191 benign, 34 malignant) were primary soft tissue tumors, 317 (259 benign, 58 malignant) were primary bone tumors, and 10 were tumor-like lesions (Table 1).

Of primary soft tissue tumors of benign nature, 102 (53%) were localized in upper extremities and 89 (47%) were localized in lower extremities including hemangioma, giant cell tumor of the tendon sheath, lipoma, synovial chondromatosis, desmoid tumor (aggressive fibromatosis), fibroma, pigmented villonodular synovitis, schwannoma, Morton neuroma, epidermal cyst, and ganglion (Tables 1, 2, 6), whereas 8 (24%) malignant soft tissue tumors were localized in upper extremity, and 26 (76%) were in lower extremity with the diagnosis of synovial sarcoma, dermatofibrosarcoma protuberans, rhabdomyosarcoma, clear cell sarcoma, Kaposi sarcoma, fibrosarcoma, adrenocortical carcinoma metastasis, and liposarcoma (Tables 1, 3, 7).

On the other hand, regarding benign primary bone tumors, 64 (25%) were localized in the upper extremity, and 195 (75%) were in the lower extremity including chondroblastoma, enchondroma, osteochondroma, aneurysmal bone cyst, osteoblastoma, simple bone cyst, osteoid osteoma, fibroxanthoma, giant cell tumor of bone, fibrous dysplasia, chondromyxoid fibroma, non-ossifying fibroma, intraosseous lipoma, fibrous cortical defect, intraosseous hemangioma, and eosinophilic granuloma (Tables 1, 4, 8). Furthermore, 14 (24%) malignant bone tumors were localized in the upper extremity, and 44 (76%) were in the lower extremity with a variety of Ewing sarcoma, osteosarcoma, chondrosarcoma, epithelioid sarcoma, multiple myeloma, solitary plasmacytoma, fibrosarcoma, and metastasis (Tables 1, 5, 9).

Of tumor-like lesions, 7 myositis ossificans, 2 Brodie abscess, and a bone cyst hydatid were managed without any recurrence (Table 10).

Regarding medical and radiation treatments, we observed chemotherapy resistance in some of Ewing sarcomas which were followed by autologous bone marrow transplantation sequentially. On the other hand, outcomes of conventional chemotherapy or radiotherapy in other tumors were successful in terms of controlling local recurrence or metastasis.

A detailed explanation regarding treatment options, recurrence, and revisions were showed in each table.

## Discussion

In the present study, we evaluated the bone and soft tissue tumors, and tumor-like lesions in a local population consisted of military personnel and their families in a tertiary military hospital. Our results seem to be an indicator for geographic distribution of musculoskeletal tumors which may be cater to an entire country.

There are few comprehensive epidemiological reports to demonstrate the incidence and diagnostic distribution of musculoskeletal system tumors in Turkey. In a large population study of 20-year data, Yuceturk et al. reported a total of 5658 musculoskeletal tumor cases to be aware of the specific rates in Aegean region (6), moreover Dabak et al. published a 25-year data of musculoskeletal tumors including 1925 cases from the central Black sea region (7). In a recent report, Ozturk et al. experienced a total of 3,133 patients with the diagnosis of musculoskeletal tumors from different regions of Turkey in their tertiary care center (8). Among those previous reports, only one presents the data of musculoskeletal oncology experience in military personnel which is carried out in our hospital 26 years ago. In this report, Gür et al. presented their 10-year experience of 420 musculoskeletal tumor patients in a tertiary military hospital (9). Their findings were consistent with the literature regarding the prevalence of bone, soft tissue tumors and metastasis in Turkey. Our study may be considered as the continuation of the Gür et al.'s study regarding the incidence of musculoskeletal tumors in military personnel and families.

There is no study of distribution of musculoskeletal tumors regarding the localization of skeleton in terms of upper and lower extremities yet. In our results, primary soft tissue tumors are most frequently seen in upper extremities than malignant soft tissue tumors, on the contrary malignant primary bone tumors are mostly seen in lower extremities than upper extremities.

Considering the prevalence of tumors, the most common benign soft tissue tumors were reported as lipoma, ganglion and giant cell tumor of tendon sheath, respectively, which was consistent with the literature (6–8). Whereas, the most common malignant soft tissue tumors have been reported as liposarcoma, pleomorphic cell sarcomas (malign fibrous histiocytomas), leiomyosarcomas, and synovial sarcomas, respectively, in a large referral population (6, 10). In our series, most common malignant soft tissue tumor was liposarcoma, then synovial sarcoma took place. Excepting pleomorphic cell sarcomas and leiomyosarcomas, the result showed consistency with the literature.

In similar epidemiological studies all around the world, it is conducted that primary benign bone tumors have higher incidence than malignant ones (11–14). In our study, benign bone tumors accounted for 47% of all our patients in consistency with those reports. Moreover, osteochondroma, simple bone cyst, and chondromas were the most common benign lesions identified in those reports. In a report it is accounted for 21.3% of all tumors, while the percentage of 28.5% among all tumors was reported in another study (12, 14). Similarly, osteochondroma, enchondroma, and simple bone cysts were the most common ones observed in upper extremity in our study, and osteochondroma accounted for 21.6% of all benign bone tumors. There is controversy related to the incidence of aneurysmal bone cyst which was found to be the fourth most common benign tumor in upper extremity. Interestingly, osteoid osteoma followed as the second most common benign bone tumor after osteochondroma, constituting a relatively high percentage of all tumors in the lower extremity. Kitsoulis reported an incidence of osteoid osteoma to represent 11% of all benign bone tumors (15), whereas it was found to be 18% of all benign tumors, predominantly in the lower extremities.

Malignant bone tumors accounted for 10% of all tumors, and 18% among all bone tumors, respectively, in this study. Among the malignant bone tumors, osteosarcoma was the most commonly encountered in the literature (6, 8, 9, 14, 16), however Sevimli reported chondrosarcoma as the most common malignant bone tumor in their locally designed studies (17). Bergovec et al. reported chondrosarcoma to be the second most common of all tumors, with its accounting of 2.9%, as Unni et al. in a study of bone malignancies, reported chondrosarcoma to be the third most common of all bone tumors, at 10.7% (14, 18). In the present study, the most common primary malignant bone tumor was osteosarcoma, with an incidence of 39% of all malignant bone tumors, predominantly in the lower extremity and consistently with the literature. Ewing sarcoma and chondrosarcoma were the second and third most common malignant bone tumors, accounted for 20 and 15%, respectively.

In the present study, the most common localizations of metastasis were found as femur and humerus, predominantly in the lower extremity. Though most common localizations of metastasis have been reported in vertebral column, surgical management has been encountered by neurosurgery clinics of our hospital. On the other hand, the most vertebral column metastasis could be treated conservatively in our clinic which would be the reason of the less common incidence of this region.

Regarding sex, male dominancy has been reported with a ratio of 1.5 for musculoskeletal tumors by Solooki et al.'s (13), whereas Solakoglu and Benzer (19) reported a male dominancy in bone tumors and female dominancy in soft tissue tumors which was supported by Ozturk et al. (8). In the current study, musculoskeletal tumors have been found more common in males than females which could be contributed to the study population of male domination.

This study gives an overview of the surgical experiences of military doctors in musculoskeletal oncology. Beyond a great experience over years in trauma surgery due to war injuries which may considered as the bad fate of the country, musculoskeletal tumor surgery has been a great part as trauma surgery. In the past, a similar study has been reported by our masters which may be determined as preliminary reports of the experience of military doctors in musculoskeletal oncology. However, development of technology in the orthopedic industry and treatment strategies have occurred in the past several years which could attribute us using custom-made implants in the management of necessary situations. Our 3D reconstruction center allowed us to manage some malignant lesions with custom-made implants. It is initially designed and constructed for reconstruction of bone defects in military personnel sustained to war-related injuries and their families, then it is adopted to other situations of dental clinic, neurosurgery and orthopedics including dental defects, cranial defects, and musculoskeletal oncologic reconstructions. On the basis of this, we have performed one custom-made tumor resection prosthesis in a young female with a diagnosis of fibrosarcoma in iliac wing which was not a possible treatment modality in our country. Further studies of this topic is warranted which could shed more light on newer changes in trends of musculoskeletal tumors, enabling creation of better, and newer strategies for diagnosing and treatment.

Furthermore, there are several studies from different regions in Turkey which were also aimed to present the geographical distribution of bone and soft tissue tumors, however the results showed that there was no any regional difference (6, 9, 19). Moreover, our results in terms of distribution and epidemiology showed parallel findings previously reported relevant published data in other developed and underdeveloped countries from all around the world (5, 11–15).

There are some limitations in this study. First this is a retrospective study of prospectively gathered data, and while collecting data we noted a 5% of lost data and wrong diagnosis (especially in tumor-like lesions) due to incomplete medical records. So, we did not comment on the incidence of tumor-like lesions in the study. Second as our clinic is not the referral center for spinal surgery, we assume the incidence of musculoskeletal tumors and metastasis affecting the spine greater than reported in this study. Third, this paper contains only data about surgically treated tumors, however some benign soft tissue and bone tumors, and tumor-like lesions are discovered accidentally and most of which do not need biopsy or surgical management. It is clear that the incidence of such lesions and tumors is much higher than reported, but there is not a method to determine this situation exactly. Last we have not enough data comparing the outcomes of chemotherapy in Ewing sarcoma and other tumors.

## Conclusions

In conclusion, though musculoskeletal disorders are rare, the principles of management should be well-known by increasing the awareness to this highly subspecialized field. In our country it is well-established by tertiary military hospital as other several referral centers, however there is still need for highly equipped oncology centers with the growing population of the world.

## Data Availability Statement

The datasets generated for this study are available on request to the corresponding author.

## Ethics Statement

The studies involving human participants were reviewed and approved by Gülhane Military Medical Academy Local Ethics Committee (2014–13).

## Author Contributions

CN and YE substantial contributions to conception and design, acquisition of data, or analysis and interpretation of data, drafting the article or revising it critically for important intellectual content, and final approval of the version to be published.

### Conflict of Interest

The authors declare that the research was conducted in the absence of any commercial or financial relationships that could be construed as a potential conflict of interest.
